# Estimating surgical probability: Development and validation of a prognostic model for patients with lumbar disc herniation treated with acupuncture

**DOI:** 10.1097/MD.0000000000036425

**Published:** 2023-12-01

**Authors:** Di Chen, Zimeng Lv, Yicheng Wu, Panfu Hao, Liu Liu, Bin Pan, Haiping Shi, Youlu Che, Bo Shen, Peng Du, Xiaohua Si, Zhongling Hu, Guorui Luan, Mingxin Xue

**Affiliations:** a Nanjing University of Chinese Medicine, Nanjing, China; b Department of Tui Na, the First Affiliated Hospital of Anhui University of Chinese Medicine, Hefei, China; c Acupuncture Rehabilitation Department, the Second Affiliated Hospital of Anhui University of Chinese Medicine, Hefei, China; d Department of Rehabilitation Medicine, Anhui NO.2 Provincial People’s Hospital, Hefei, China; e Department of Tui Na, Anhui Provincial Hospital of Integrated Chinese and Western Medicine, Hefei, China; f Acupuncture Rehabilitation Department, Traditional Chinese Hospital of Luan, Luan, China; g The First Clinical Medical College of Nanjing University of Chinese Medicine, Nanjing, China.

**Keywords:** acupuncture, lumbar disc herniation, nomogram

## Abstract

Lumbar disc herniation (LDH) is a common cause of pain in the lumbar spine and legs. While acupuncture has become the primary conservative treatment for LDH, some patients experience treatment failure and require surgery, causing substantial concern for clinicians. We developed an effective personalized clinical prediction model to identify the independent risk factors associated with acupuncture failure in patients with LDH. Our model aimed to predict the probability of surgery within 6 months of acupuncture failure in patients with LDH. A total of 738 patients with LDH who underwent acupuncture at 4 Chinese hospitals between January 2019 and October 2021 were selected. The patients were divided into training (n = 496) and validation (n = 242) cohorts. Seven predictive variables, including smoking, Oswestry Disability Index (ODI) score, lower-limb herniation, disc herniation type, lumbar spinal stenosis, lumbar lateral recess stenosis, and acupuncture frequency, were selected as risk factors using least absolute shrinkage and selection operato (LASSO) regression. A prediction model was developed using multivariate logistic regression analysis and a nomogram was constructed. The model exhibited good discrimination, with an area under the ROC curve (AUC) of 0.903 for the development cohort and 0.899 for the validation cohort. The Hosmer-Lemeshow goodness-of-fit test was a good fit for both cohorts (*P* = .956 for the development cohort; *P* = .513 for the validation cohort). Decision curve analysis (DCA) demonstrated that the threshold probabilities for the 2 cohorts ranged from > 4% and 5–95%, respectively. Therefore, the prediction model had a good net benefit. The nomogram established in this study, incorporating 7 risk factors, demonstrated a good predictive ability. It could predict acupuncture failure in LDH patients and the risk of surgery within 6 months, enabling physicians to conduct individualized treatment measures.

## 1. Introduction

Lumbar disc herniation (LDH) is characterized by lumbar and lower extremity radiating pain resulting from lumbar disc degeneration or injury, rupture of the annulus fibrosus, and protrusion of the nucleus pulposus, which irritates or compresses the nerves.^[1]^ In China, the incidence of LDH is increasing annually owing to factors such as an aging population and a fast-paced lifestyle. This trend shows a younger age at onset and imposes a significant social and economic burden, severely impacting people’s quality of life.^[2,3]^ Current treatment for LDH is broadly divided into “conservative treatment and “surgical treatment.”^[4]^

The primary surgical indication for LDH is a radiculopathy with neurological deficits. Despite advancements in surgical techniques, whether surgical resection provides a “meaningful” benefit for all LDH patients remains unclear. It is widely accepted that due to the favorable clinical course of LDH, conservative treatment should be the first-line treatment in the absence of severe or progressive neurological deficits and intractable pain. Patients typically undergo conservative treatment for at least 6 months; and surgery is only advised if conservative methods fail.^[5–7]^ Acupuncture is a widely accepted among the various conservative therapy for LDH.^[8]^ Extensive research has proved the long-term efficacy and safety of acupuncture, effectively reducing the need for surgery.^[9,10]^ Despite these positive outcomes, some patients did not experience improvement with acupuncture and resorted to surgery. This delay can result in missed opportunities for timely surgical intervention, leading to a prolonged disability, high treatment costs, and chronic pain. Therefore, it is crucial to further explore and determine the optimal choice between conservative and surgical treatment at an early stage.^[11,12]^

Therefore, identifying patients with LDH who may not be suitable for conservative treatment could assist in developing patient-specific care strategies. Nomograms have recently emerged as statistical visualization tools for predicting disease onset, progression, prognosis, and survival.^[13]^ Most scholars at home and abroad have explored the research on the prognosis of LDH patient treatment and have established visualization prediction models.^[14–16]^ However, most of the above clinical prediction models explore the prognosis of postoperative patients, which limits the clinical application to some extent. In view of adverse outcomes, early warning and decision-making are needed in clinical practice. To our knowledge, few studies have focused on predicting the probability of surgery within 6 months of ineffective acupuncture for LDH. Consequently, we constructed a predictive model that identified independent predictors of conservative treatment and failed to determine which individuals were most likely to benefit from LDH surgery. This study aimed to develop and validate a nomogram model using a large multicenter database to predict the probability of failure within 6 months of surgery in patients with acupuncture-treated LDH.

In this study, through reviewing the literature and according to the purpose of this study, we selected commonly used and easily accessible clinical data, screened the best risk factors and constructed a model using the least absolute shrinkage and selection operator (LASSO) regression method, visualized the model using a nomogram, and finally externally validated the model, predicted the risk of failure to take surgical treatment for patients with LDH treated with acupuncture, provided individualized treatment plans for patients, and assisted clinicians in making the best clinical decisions.

## 2. Materials and methods

### 2.1. Study population

**Figure 1.**
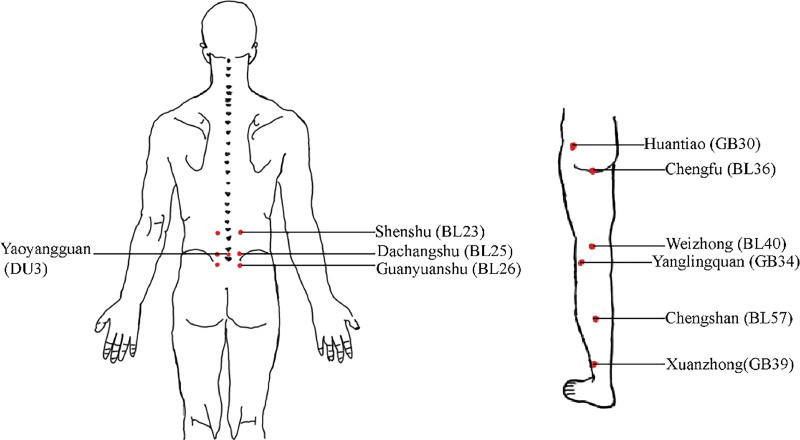
Schematic representation of needle acupoints.

This study aimed to establish a prospective longitudinal registry of patients who received acupuncture for LDH at 4 Chinese hospitals (286 individuals from the First Affiliated Hospital of Anhui University of Chinese Medicine; 210 individuals from Anhui No.2 Provincial People’s Hospital; 139 individuals from Anhui Provincial Hospital of Integrated Chinese and Western Medicine; and 103 individuals from Traditional Chinese Hospital of Luan) between January 2019 and October 2021. The registry captured information on patients who underwent acupuncture for LDH after hospital admission. All 4 facilities used the same treatment protocol. Acupuncture point selection followed the guidelines outlined in the 10th Edition of the National Higher Schools of Traditional Chinese Medicine Planning Textbook “Acupuncture and Moxibustion” for treating LDH. A semi-standardized acupuncture protocol was developed by combining the textbook method with clinical experience and expert opinions, incorporating distant and nearby acupuncture points.^[17]^ Acupoints on problematic meridians were used in this group as part of a semi-standardized treatment. The treatment plan included 7 obligatory acupoints and 5 adjunct acupoints (Fig. 1). The obligatory acupoints included bilateral Shenshu (BL23), Dachangshu (BL25), Guanyuanshu (BL26), and Yaoyangguan (DU3). The adjunct acupoints used were Huantiao (GB30), Yanglingquan (GB34), Xuanzhong (GB39), Chengfu (BL36), Weizhong (BL40), and Chengshan (BL57) acupoints. Filiform needles measuring 0.3 × 40.0 mm and 0.3 × 100.0 mm (Suzhou Tianxie Acupuncture Equipment Co., Ltd., Suzhou, China) were used in this study. Direct acupuncture using a needle depth of 25–40 mm was applied to bilateral Shenshu (BL23), Dachangshu (BL25), Guanyuanshu (BL26), Yaoyangguan (DU3), Yanglingquan (GB34), Xuanzhong (GB39), Chengfu (BL36), and Chengshan (BL57); 40 to 60 mm needle depth to Chengfu (BL36); and 70 to 90 mm needle depth to Huantiao (GB30), targeting a noticeable radiating acupuncture sensation at the bottom of the foot. The twisting, lifting, and thrusting techniques were performed gently and evenly 3 times to achieve a de qi sensation.

The treatment groups received acupuncture for 4 weeks, 2 to 3 times per week for 30 minutes each. The acupuncture frequency was chosen based on patient preference and acupuncturist experience. The inclusion criteria were as follows: meeting the diagnostic criteria for LDH,^[18]^ patients of either sex and between 18 and 70 years of age, and patients who provided informed consent and voluntarily received acupuncture. The exclusion criteria were as follows: previous history of LDH surgery; severe liver or kidney dysfunction, coagulation dysfunction, or mental disorders; Sequestration-Type LDH or lumbar spondylolisthesis degree II or above; lumbar fracture; receipt of other non-surgical treatments during the study period, including medications and noninvasive interventions such as epidural steroid injections or physical therapy; and severe neurological deficits.

### 2.2. Potential predictive variables

By conducting a literature review and considering the study objectives, 15 predictors were screened.^[19–22]^ The basic characteristics and imaging findings of the patients were prospectively collected from each hospital care system. Potential predictive variables obtained at hospital admission included age, sex, body mass index (BMI), smoking (defined as daily consumption of more than 1 cigarette for a continuous or cumulative period of 6 months, persisting until 1 week before treatment), duration, Oswestry Disability Index (ODI) at admission, degree of positive straight leg raise test, lower limb muscle strength, lower limb sensation, disc herniation level and type, lumbar spondylolisthesis, lumbar spinal stenosis, and lumbar lateral recess stenosis. Acupuncture frequency predictors were collected after treatment. The endpoint of this study was to determine whether acupuncture was effective in improving the symptoms and reducing the probability of surgery in patients with LDH. Patients with acupuncture-treated LDH were followed up for 6 months for surgery, and the endpoint event (independent variable) was assigned as “No Surgery = 0” and “Surgery = 1.”

### 2.3. Training and validation cohorts

The 4 institutions in this study were divided into 2 groups to develop a nomogram and perform independent external validations. The training cohort (n* *= 496) was assigned to the First Affiliated Hospital of the Anhui University of Chinese Medicine and Anhui No.2 Provincial People’s Hospital, whereas the validation cohort (n* *= 242) was assigned to the remaining 2 institutions.

### 2.4. Follow-up and study endpoints

Following the relevant literature, 3 to 6 months of non-surgical treatment is recommended for LDH without cauda equina syndrome. Aligning with the objectives of this study, patients with acupuncture-treated LDH were followed up for 6 months. After hospital discharge, all the patients were regularly monitored at each participating institution. The monitoring strategy involved telephone follow-up every 2 months following the completion of treatment. The study endpoint was acupuncture failure in patients with LDH, leading to surgical intervention within 6 months. Despite effective conservative treatment, recurrence within the 6-month treatment period was considered a negative outcome.

### 2.5. Sample size

The effective sample size in the prediction study was determined by the number of endpoint events, with at least 10 endpoint events per variable (EPV) to ensure precision.^[23]^ The proportion of patients with LDH receiving conservative treatment varies between 20% and 50% of those undergoing surgery, depending on symptom severity and herniation type.^[24]^ To accommodate 10 or fewer predictors in the final multivariate logistic regression model, a minimum training cohort of 200 patients was required. Our sample size and the number of endpoint events surpassed the EPV recommendation, ensuring robust and reliable predictions.

### 2.6. Ethics statement

This study was approved by the institutional review boards of 4 institutions (First Affiliated Hospital of Anhui University of Chinese Medicine and Anhui Provincial Hospital of Integrated Chinese and Western Medicine, 2018zryb25; Anhui NO.2 Provincial People’s Hospital, ztn20191016; Traditional Chinese Hospital of Luan, LAZD011). Informed consent was obtained from all the patients before enrollment in the study. This study was conducted in accordance with the principles of the Declaration of Helsinki and its subsequent amendments.

### 2.7. Statistical analysis

In this study, clinical data with missing values below 20% were imputed using multiple imputations (MI) in R software, employing the “mice” package. Numerical features were imputed using predictive mean matching, binary variables using logistic regression, and factor features using Bayesian polytomous regression. Categorical data are expressed as composition ratios, and the χ^2^ test was used to compare the 2 groups. The LASSO regression technique was used to select the optimal predictive features in the training set. The optimal parameter (λ) in the LASSO model was selected through minimum standard selection using 10-fold cross-validation, with partial likelihood deviation as the Y-axis and log(λ) as the X-axis, with the minimum λ value and the minimum λ value of 1SE plotted as the dashed vertical line at the optimal value, with the minimum λ value of 1SE as the optimal value of the model. The optimal diagnostic model, based on the minimal Akaike information criterion, was selected to determine the variables to be included in the model. The model was constructed through screening of predictors, and the regression coefficients of the independent variables were used to establish an individualized prediction model for the outcome of acupuncture-treated LDH.

This study conducted external validation using a validation set to evaluate the prediction model based on 3 key aspects: discrimination, calibration, and net benefit. Discrimination was assessed using the area under the curve of the receiver operating characteristic (ROC) curve, which measured the ability of the prediction model to distinguish between LDH patients who received acupuncture and subsequently underwent surgery versus those who did not.^[25]^ Calibration was assessed using calibration curves and the Hosmer-Lemeshow goodness of fit test, which examined the agreement between predicted and observed probabilities. Clinical validity was assessed using decision curve analysis (DCA).^[26–28]^

SPSS software (version 26.0, SPSS Inc., Chicago, IL) and R version 4.1.3 were used for data analysis. Statistical significance was defined as a two-tailed *P* value of *P* < .05.

## 3. Results

### 3.1. Patient characteristics

**Figure 2.**
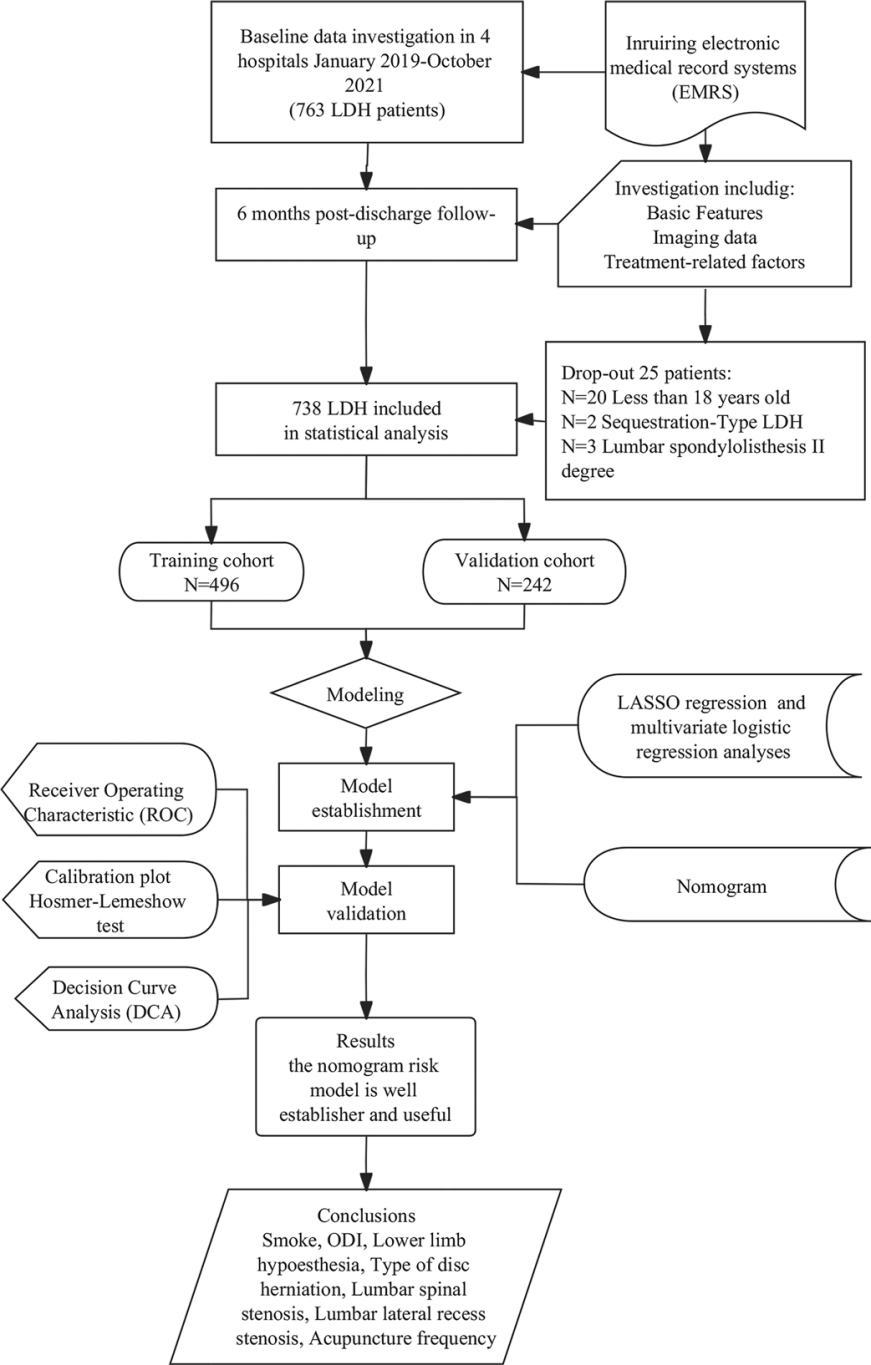
Flow chart of patient inclusion.

Two patients with Sequestration-Type LDH and 3 with lumbar spondylolisthesis II degrees were excluded from the 763 patients who underwent acupuncture for LDH between January 2019 and October 2021. The final analysis cohort comprised 738 patients with a 6-month follow-up:141 underwent surgery, while 597 did not. Among these patients, 496 (67.2%) were assigned to the training cohort and 242 (32.8%) to the validation cohort (Fig. 2). Baseline data comparison revealed that the training and validation cohorts were similar in terms of clinical characteristics, including general patient condition, imaging examinations, and treatment-related data (all *P *> .05). To facilitate the clinical application of continuous variables, they were categorized based on the understanding that changes in age and duration units had a limited impact on outcome risk. Age equivalents were divided into “<45,” “45–59,” and “60,” and duration was divided into “<60” and “≥60” years. BMI, ODI, and the degree of positive straight leg raise test results were grouped based on their professional significance. The other independent variables were defined as categorical variables (Table 1).

**Table 1:** Clinical characteristics of the study population.

Factors level		Whole cohort (n = 738)	Training cohort (n = 496)	Validation cohort (n = 242)	*P* value
Age, n (%)	<45	163 (22.1)	107 (21.6)	56 (23.1)	.839
	45–59	359 (48.6)	241 (48.6)	118 (48.8)	
	≥60	216 (29.3)	148 (29.8)	68 (28.1)	
Gender, n (%)	Female	419 (56.8)	285 (57.5)	134 (55.4)	.591
	Male	319 (43.2)	211 (42.5)	108 (44.6)	
BMI, n (%)	Obese	34 (4.6)	25 (5.0)	9 (3.7)	.118
	Normal weight	583 (79.0)	399 (80.4)	184 (76.0)	
	Overweight	121 (16.4)	72 (14.5)	49 (20.2)	
Smoking, n (%)	No	576 (78.0)	396 (79.8)	180 (74.4)	.093
	Yes	162 (22.0)	100 (20.2)	62 (25.6)	
ODI, n (%)	Minimal disability	9 (1.2)	8 (1.6)	1 (0.4)	.473
	Moderate disability	143 (19.4)	92 (18.5)	51 (21.1)	
	Severe disability	394 (53.4)	267 (53.8)	127 (52.5)	
	House-bound	192 (26.0)	129 (26.0)	63 (26.0)	
Duration (mo), n (%)	<60	574 (77.8)	384 (77.4)	190 (78.5)	.737
	≥60	164 (22.2)	112 (22.6)	52 (21.5)	
Degree of positive straight leg raise test (°), n (%)	<60	629 (85.2)	422 (85.1)	207 (85.5)	.870
	≥60	109 (14.8)	74 (14.9)	35 (14.5)	
Leg weakness, n (%)	No	593 (80.4)	389 (78.4)	204 (84.3)	.060
	Yes	145 (19.6)	107 (21.6)	38 (15.7)	
Lower limb hypesthesia, n (%)	No	570(77.2)	387 (78.0)	183 (75.6)	.465
	Yes	168(22.8)	109 (22.0)	59 (24.4)	
Level of disc herniation, n (%)	1	140 (19.0)	95 (19.2)	45 (18.6)	.697
	2	272 (36.9)	178 (35.9)	94 (38.8)	
	3	233 (31.6)	162 (32.7)	71 (29.3)	
	4	76 (10.3)	48 (9.7)	28 (11.6)	
	5	17 (2.3)	13 (2.6)	4 (1.7)	
Type of disc herniation, n (%)	Bulge	61 (8.3)	42 (8.5)	19 (7.9)	.361
	Herniation	627 (85.0)	416 (83.9)	211 (87.2)	
	Prolapse	50 (6.8)	38 (7.7)	12 (5.0)	
Lumbar spondylolisthesis, n (%)	No	689 (93.4)	459 (92.5)	230 (95.0)	.200
	Yes	49 (6.6)	37 (7.5)	12 (5.0)	
Lumbar spinal stenosis, n (%)	No	560 (75.9)	370 (74.6)	190 (78.5)	.243
	Yes	178 (24.1)	126 (25.4)	52 (21.5)	
Lumbar lateral recess stenosis, n (%)	No	632 (85.6)	434 (87.5)	200(82.6)	.075
	Yes	106 (14.4)	62 (12.5)	42 (17.4)	
Acupuncture frequency (10/month), n (%)	No	199 (27.0)	135 (27.2)	64 (26.4)	.825
	Yes	539 (73.0)	361 (72.8)	178 (73.6)	

### 3.2. Predictors and predictive modeling of surgery rate within 6 months after treatment

**Figure 3.**
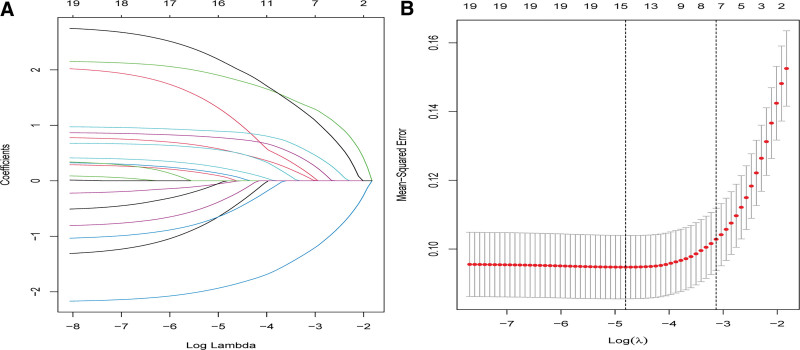
(A) Optimal parameter (λ) selection in the LASSO model, with the optimal tuning parameter logλ in the horizontal coordinate and the regression coefficient in the vertical coordinate; (B) Distribution of LASSO coefficients for the 15 factors, with the optimal tuning parameter logλ in the horizontal coordinate and the binomial deviance in the vertical coordinate.

**Figure 4.**
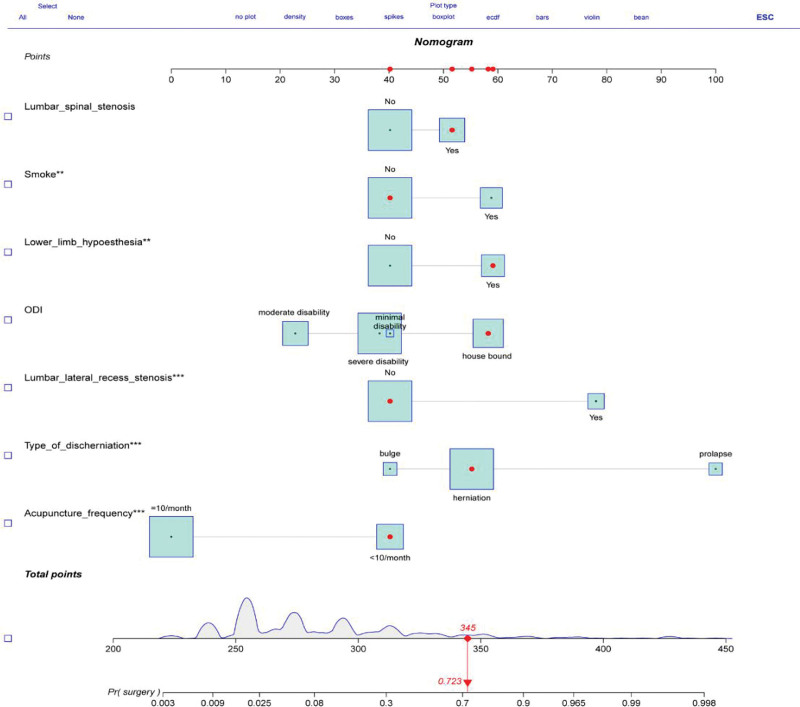
Nomogram for predicting the probability of surgery in LDH patients within 6 months of receiving acupuncture.

**Figure 5.**
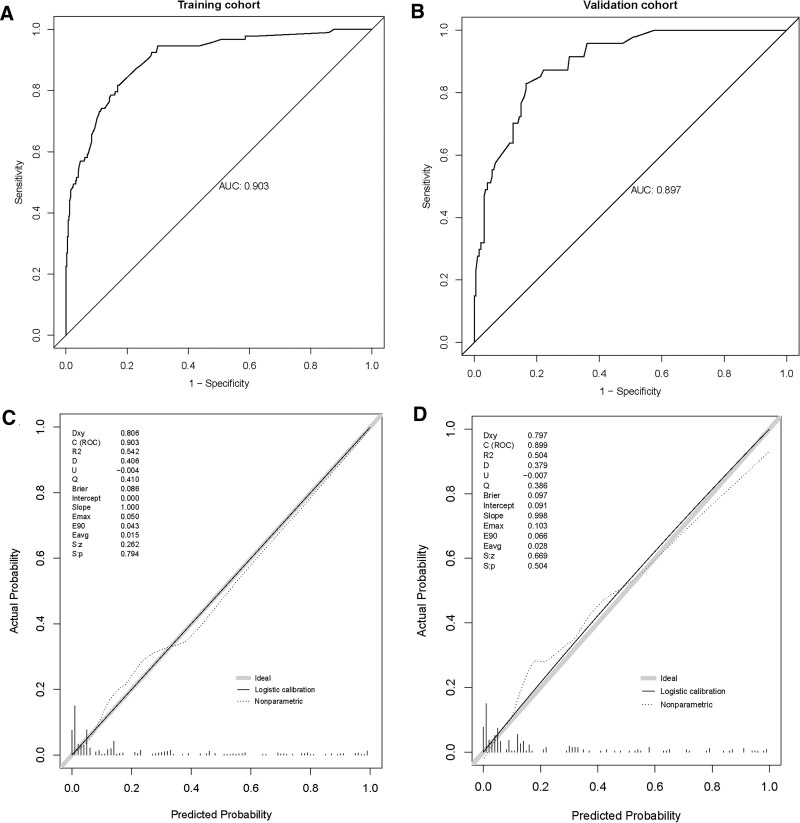
Receiver operating characteristic (ROC) curves. (A) ROC curve in the training cohort; (B) ROC curve in the validation cohort; (C) calibration plots in the training cohort; (D) calibration plots in the validation cohort of the model for the probability of surgery in LDH patients within 6 months after receiving acupuncture. LDH = lumbar disc herniation.

Based on the baseline characteristics, physical examination, imaging, and treatment of patients in the training cohort, 15 predictive variables were downscaled using LASSO regression to identify the most influential factors on surgical probability (Fig. 3). Seven predictive variables were selected as the most influential. The screened predictive variables included smoking status, ODI score, lower-extremity herniation, type of disc herniation, lumbar spinal stenosis, lumbar lateral recess stenosis, and acupuncture frequency. A multivariate logistic regression prediction model was constructed using the 7 most influential predictors as independent variables and assessing whether patients with LDH received acupuncture within 6 months after discharge as the dependent variable (Table 2). Moreover, we constructed a nomogram prediction model for surgery within 6 months of LDH acupuncture (Fig. 4). Each variable was assigned a specific score. The total score on the total scale was obtained by summing all the scores, and a vertical line was drawn downward to predict the probability of surgery within 6 months after treatment. For example, a patient with low back pain without a smoking history (40 points), an ODI score of 4 (59.5 points), lower limb hypesthesia on physical examination (58.5 points), LDH detected on MRI (55 points), lumbar spinal stenosis (52 points), absence of lumbar lateral recess stenosis (40 points), and receiving acupuncture less than 10 times per month (40 points). The cumulative score for each predictor was 345, which corresponded to a predicted surgical risk of 0.723 (72.3%). Based on the predicted probabilities, the patient exhibited a high probability of surgery. This calculated value is significant for treatment planning and patient counseling in decision-making. The model showed commendable accuracy in predicting the probability of surgery within the 6-month treatment period, with a C-index of 0.903 (95% CI: 0.869–0.938). The model was well calibrated for risk estimation, as evidenced by the Hosmer–Lemeshow test chi-square statistic of 3.20 (*P *= .956; Fig. 5A and C).

**Table 2:** Results of multivariate logistic regression analysis of 7 clinical characteristics screened by LASSO regression.

Factors level		Β	SE	OR (95% CI)	*P* value
Smoking	Yes vs No	1.01	0.36	2.75 (1.37–5.52)	.004
ODI	Moderate disability vs Minimal disability	−0.97	1.15	0.39 (0.04–3.69)	.410
	Severe disability 3 vs Minimal disability	−0.11	1.05	0.90 (0.11–7.11)	.921
	House-bound vs Minimal disability	0.98	1.06	2.67 (0.33–21.41)	.356
Lower limb hypesthesia	Yes vs No	1.03	0.34	2.80 (1.45–5.41)	.002
Type of disc herniation	Herniation vs Bulge	0.82	0.81	2.27 (0.46–11.14)	.315
	Prolapse vs Bulge	3.26	0.94	25.94 (4.14–162.54)	.001
Lumbar spinal stenosis	Yes vs No	0.62	0.34	1.86 (0.96–3.61)	.067
Lumbar lateral recess stenosis	Yes vs No	2.06	0.05	7.84 (3.55–17.32)	<.001
Acupuncture frequency	Yes vs No	−2.19	0.33	0.11 (0.06–0.22)	<.001
Constant		−2.49	1.35	0.08	.065

### 3.3. Validation and risk grouping based on the nomogram model

**Figure 6.**
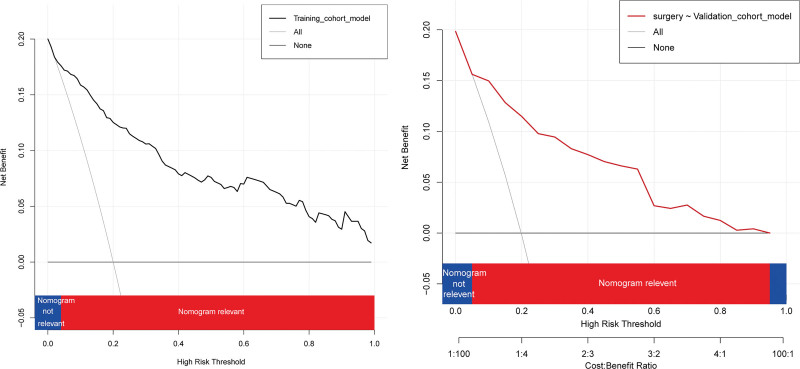
Decision curve analysis (DCA) for predicting surgery in LDH patients. (A) training cohort; (B) validation cohort. LDH = lumbar disc herniation.

External validation was used to assess model performance in terms of discrimination and calibration ability. The nomogram demonstrated consistent performance when applied to the validation cohort, with a C-index of 0.899 (95% CI: 0.855–0.942), predicting the probability of surgery for patients within 6 months of receiving acupuncture (Fig. 5B). The Hosmer-Lemeshow chi-square test confirmed the calibration power (8.21, *P *= .513). The calibration plot demonstrated a good fit in the validation cohort, indicating agreement between the predicted probability of surgery within 6 months of acupuncture and actual observations (Fig. 5D). DCA demonstrated the same significant net benefit in the training and validation cohorts (Fig. 6A and B). The threshold probabilities, plotted on the x-axis, represent the range of appropriate risk probabilities (identified beforehand) for guiding treatment decisions compared with the default strategies of “treatment for all” and “treatment for no one.” The net benefit of using the column line plot to predict the probability of failure within 6 months of surgery in acupuncture-treated LDH patients was significantly higher compared to the “no intervention” and “full intervention” cohorts. The threshold probabilities for the training and validation cohorts were > 4% and 5% to 95%, respectively, suggesting the clinical applicability of the nomogram. We attempted to develop a predictive model to identify independent predictors of acupuncture failure to determine which LDH patients would benefit the most from surgery. Our nomogram is useful for clinical decision-making as it considers various treatment measures, including surgical and non-surgical treatments.

## 4. Discussion

LDH is a common condition seen in orthopedic clinics and a leading cause of low back pain, significantly affecting the quality of life and daily work activities of patients.^[11]^ Nonsurgical treatment is often used to control pain and restore bodily function.^[29]^ However, some patients find non-steroidal anti-inflammatory drugs ineffective, and prolonged medication use may cause adverse effects including headaches, nausea, and abdominal pain.^[30,31]^ Acupuncture has emerged as a preferred conservative treatment option attributed to its notable benefits of “fast efficacy and minimal adverse effects.”^[32]^ Previous studies have shown that acupuncture can enhance blood circulation and oxygen supply to the cauda equina, nerve root, and sciatic nerve, promoting nerve recovery and symptom improvement.^[33]^ Simultaneously, acupuncture can modulate the function of the endogenous pain regulation network,^[34]^ providing rapid analgesic effects in patients.^[35]^

Acupuncture has shown remarkable effectiveness in treating LDH; however, conservative treatment is ineffective in some patients, as the number of cases increases. Surgical treatment is a major clinical concern. Combined with the relevant data, 20% to 50% of LDH cases eventually require surgery because of conservative treatment failure. Therefore, identifying the risk factors for treatment failure in patients with LDH is crucial to provide proactive preventive measures.^[24]^ The likelihood of conservative treatment in patients with LDH at the outset must be predicted using this method. Nomograms, which integrate multiple important factors into a visual graphical representation of a mathematical model, offer personalized risk assessment and have become reliable and convenient tools for quantifying risk. Nomograms facilitate disease management and related clinical decision-making.^[36]^

This study identified smoking, ODI score, lower limb hypesthesia, disc herniation type, lumbar spinal stenosis, lumbar lateral recess stenosis, and acupuncture frequency as predictive variables affecting surgery after conservative treatment failure in patients with LDH. The results revealed that smoking was associated with poor clinical efficacy of acupuncture for LDH treatment. Many studies have reported a relationship between smoking and lower back pain, indicating that smoking impairs oxygen transport, carboxyhemoglobin formation, vasoconstriction, and fibrinolytic activity disturbance, resulting in impaired intervertebral disc nutrition and reduced blood flow. These factors increase the risk of low back pain.^[37,38]^ The ODI score, known for its stability and reliability, can be used as a reference indicator to assess comprehensive rehabilitation outcomes in patients with low back pain. Higher baseline ODI scores are predictive of LDH outcomes.^[39]^ Local skin sensory loss in the lower extremities affects the overall outcomes of patients with LDH. Most patients exhibit nerve damage at L4–5, or S1 before treatment, as these nerve roots innervate sensory function in the lower extremities. Once sensory function is impaired, achieving full and prompt restoration through acupuncture is challenging.^[40]^ Disc herniation is another crucial factor that affects patient recovery and warrants clinical attention. A larger disc herniation causes greater nerve root compression and more severe clinical symptoms. Additionally, conservative treatment failure showed a significant and independent correlation with lumbar spinal stenosis, as the proportion of LDH combined with spinal stenosis was > 40%. Degenerative changes in the intervertebral discs, ligaments, and synovial joints cause normal spinal canal narrowing, and reduction in the internal diameter of the nerve root canal is the primary cause of secondary lumbar stenosis. The study results also identified lumbar lateral recess stenosis as a risk factor. It is anatomically located between the posterior edge of the disc and the ventral aspect of the superior articular eminence, and the space between the superior edge of the inferior vertebral body and the superior edge of the pedicle, which serves as a nerve root channel. When the nerve root in lumbar lateral recess stenosis is compressed due to disc herniation, ligamentum flavum thickening, and superior articular eminence hyperplasia, corresponding clinical symptoms are produced. Unfortunately, this type of acupuncture shows poor efficacy, often necessitating surgical treatment.^[41]^ The foundation of this investigation was acupuncture frequency and its treatment served as an additional independent risk factor. Acupuncture, a non-pharmacological treatment for various painful conditions, is effective and safe for pain relief. Acupuncture frequency is an important factor in determining its efficacy, and its mechanism of action may be related to its cumulative effect.^[42]^

This study used a nomogram model to predict acupuncture failure to undergo surgical treatment in patients with LDH based on the screening of predictive variables. A nomogram can be a good way to differentiate between patients with LDH who are at high versus low risk of failing acupuncture treatment to take surgical treatment within 6 months. To ensure prediction accuracy, the model was tested for predictive efficacy using an external validation method, which yielded an area under the ROC curve of 0.903 (95% CI: 0.869–0.938) and 0.899 (95% CI: 0.855–0.942) for the training and validation cohorts, respectively. The calibration curves for both cohorts fitted well with the standard curve, demonstrating the good predictive accuracy of the established model. Meanwhile, the DCA curves for both cohorts indicated improved clinical utility. The nomogram model can reflect the degree of influence of risk factors in patients with LDH who failed conservative treatment and underwent surgery. It has advantages of ease of use and intuitive continuity. The predicted probability of surgery for patients with LDH can be obtained by converting the patient indices into a functional relationship, thus completing an individualized and quantitative prediction of the patient risk profile. Many scholars at home and abroad have explored the study of treatment prognosis of LDH patients and have established visualization prediction models.^[14–16]^ However, most of the above clinical prediction models are single-center, exploring the postoperative prognosis of LDH patients. For example, one study retrospectively collected clinical data related to LDH patients treated with tubular microdiscectomy technique, and constructed and validated a prediction model for predicting the rate of improvement in the treatment of LDH patients with tubular microdiscectomy at 1 year postoperatively.^[14]^ There are also studies aiming to construct and validate a nomogram to predict residual low back pain after percutaneous endoscopic lumbar discectomy.^[15]^ Our study focused on the probability of surgery within 6 months after ineffective acupuncture treatment for LDH patients. The model can help physicians provide patients with realistic information and reasonable expectations of treatment outcomes. The prognostic model designed in this study has good discriminative, corrective, and clinical efficacy, and contains only 7 predictors, is simple and convenient to operate, and can be widely used in the daily clinical treatment of LDH patients. In addition, the current nomogram was derived from hospital databases of different sizes, and 4 participating physicians collected patient data from initial cases.

Therefore, tertiary and general hospitals can adopt the current nomogram widely. However, this study had several limitations. First, the MI approach to missing data in this study may introduce a selection bias, which requires more prospective studies to validate the findings. Second, the model includes fewer risk factors. Therefore, future validation studies should consider additional risk factors to improve predictive power. Finally, the sample size was determined according to the 10 EPV rule of thumb, because there is no uniform standard. Since this is controversial, we acknowledge the importance of paying more attention to the appropriateness of the sample size in future research, aligning with the latest guidelines.

## 5. Conclusion

In summary, smoking, ODI score, lower limb herniation, disc herniation type, lumbar spinal stenosis, lumbar lateral recess stenosis, and acupuncture frequency were predictive variables affecting the ineffectiveness of acupuncture and surgery in patients with LDH. Healthcare professionals can use it to provide predictive information for appropriate counseling, optimization, risk adjustment assessment, and treatment-related decisions to avoid “futile” acupuncture in LDH, thus further improving the prognosis of LDH patients.

## Acknowledgments

We appreciate the efforts of all the patients enrolled in this study. We thank the freescience editorial team for linguistic assistance and pre-submission expert reviews.

## Author contributions

**Conceptualization:** Di Chen, Zimeng Lv, Yicheng Wu, Panfu Hao, Mingxin Xue.

**Data curation:** Bo Shen, Peng Du, Zhongling Hu, Guorui Luan, Xiaohua Si.

**Formal analysis:** Youlu Che.

**Investigation:** Bin Pan, Haiping Shi.

**Validation:** Liu Liu.
